# Reversible Oxygen Sensing Based on Multi-Emission Fluorescence Quenching

**DOI:** 10.3390/s20020477

**Published:** 2020-01-15

**Authors:** Efe Armagan, Shankar Thiyagarajan, Kongchang Wei, Akin Gursoy, Giuseppino Fortunato, Esther Amstad, René Michel Rossi, Claudio Toncelli

**Affiliations:** 1 Empa, Swiss Federal Laboratories for Materials Science and Technology, Laboratory for Biomimetic Membranes and Textiles, Lerchenfeldstrasse 5, CH-9014 St. Gallen, Switzerland; efe.armagan@empa.ch (E.A.); shankar.nanotechnology@gmail.com (S.T.); kongchang.wei@empa.ch (K.W.); akin.gursoy@empa.ch (A.G.); giuseppino.fortunato@empa.ch (G.F.); rene.rossi@empa.ch (R.M.R.); 2 Institute of Materials, Ecole Polytechnique Fédérale de Lausanne (EPFL), CH-1015 Lausanne, Switzerland; esther.amstad@epfl.ch

**Keywords:** optical oxygen sensing, fluorescence-based oxygen quenching, multi-emission, carbon nanodots, molecular fluorophores

## Abstract

Oxygen is ubiquitous in nature and it plays a key role in several biological processes, such as cellular respiration and food deterioration, to name a few. Currently, reversible and non-destructive oxygen sensing is usually performed with sensors produced by photosensitization of phosphorescent organometallic complexes. In contrast, we propose a novel route of optical oxygen sensing by fluorescence-based quenching of oxygen. We hereby developed for the first time a set of multi-emissive purely organic emitters. These were produced through a one-pot hydrothermal synthesis using p-phenylenediamine (PPD) and urea as starting materials. The origin of the multi-emission has been ascribed to the diversity of chemical structures produced as a result of oxidative oligomerization of PPD. A Bandrowski’s base (BB, i.e., trimer of PPD) is reported as the main component at reaction times higher than 8 h. This indication was confirmed by electrospray-ionization quadrupole time-of-flight (ESI-QTOF) and liquid chromatography-mass spectrometry (LC-MS) analysis. Once the emitters are embedded within a high molecular weight poly (vinyl alcohol) matrix, the intensities of all three emission centers exhibit a non-linear quenching provoked by oxygen within the range of 0–8 kPa. The detection limit of the emission centers are 0.89 kPa, 0.67 kPa and 0.75 kPa, respectively. This oxygen-dependent change in fluorescence emission is reversible (up to three tested 0–21% O_2_ cycles) and reproducible with negligible cross-interference to humidity. The cost-effectiveness, metal-free formulation, cross-referencing between each single emission center and the relevant oxygen range are all appealing features, making these sensors promising for the detection of oxygen, e.g., in food packaged products.

## 1. Introduction

Oxygen participates in several biological processes, including food spoilage or in plant, animal and human metabolism [1,2]. It is, therefore, crucial to develop a simple sensing method to detect local changes in oxygen concentration in a reversible and non-destructive fashion (e.g., without consuming the analyte during the measurement).

In this respect, optical sensing is by far the most appealing system for oxygen monitoring, since it can be reversible and miniaturized for detecting local concentration shifts. Photoemitters exhibiting oxygen sensitivity mostly undergo quenching of long-lived emissive states, such as phosphorescence and delayed fluorescence, provoked by energy transfer with oxygen [3]. These emission modes can only be triggered by a strong spin-orbit coupling (SOC) to enable the otherwise quantum-mechanically forbidden transition from the excited singlet to excited triplet state [4]. As for now, specific organic ligands coordinated to heavy metal ions, such as Pd(II), Pt(II) or Ru(II), represent the exclusive commercial solution to trigger oxygen sensitivity [5,6,7,8]. On the other hand, several applications, such as food quality assessment, require the use of disposable oxygen sensors that have to be mass-produced with alternatives that are more cost-effective.

The development of metal-free oxygen sensors with relevant detection windows for monitoring oxygen in a physiological oxygen range is still an unmet challenge.

Purely organic emitters can display room-temperature phosphorescence (RTP) when directly connecting heteroatoms to aromatic moieties (bromo-aldehydes [9], di-ketones [10,11], di-acids and di-esters [12]) and at the same time inhibiting their internal molecular motions. This can be induced, for example, by crystallization-enhanced RTP [11,12], aggregation-induced intersystem crossing [13], encapsulation within rigid cages [14], or the rigidification of the emission centers by non-covalent [9] and covalent [15] interactions with a matrix. However, the complexity of the synthetic pathways to achieve the desired oxygen sensitivity precludes the viability of these emitters in terms of commercial exploitation.

An interesting twist of this strategy is offered by embedding bottom-up assembled carbon nanostructures (e.g., carbon nanodots, CND) in hydrogen bonding matrices, such as poly(vinyl alcohol) (PVA) [16,17], polyurethane [18] and potash alum [19], by establishing covalent cross-linking with the matrix [20,21] or by encapsulating the optical probes within rigid mesoporous cages [22,23].

In particular, red-emissive RTP CNDs can be simply obtained by hydrothermal synthesis of aromatic di-amines [24,25] and encapsulation within a poly(vinyl alcohol) (PVA) matrix [26]. However, their phosphorescence is rather long, in the order of several milliseconds, which translates into highly sensitive oxygen sensors, only applicable for extremely hypoxic environments.

Alternatively, fluorescence-based quenching using aromatic amines, such as β-carbolines [27] and acridines [28] has been demonstrated as a promising strategy for oxygen sensing. This peculiar sensing mechanism is related to the formation of charge-transfer complexes between the contact radical ion pair state ^3^(^2^M^•+^,2O^2•−^) when mixed with the locally excited (LE) states [29], however, it is not very well understood and remains unexplored in the literature.

Hereby, we demonstrate that an ensemble of hydrothermal products from urea and p-phenylenediamine gives rise to multi-emissive oxygen sensors once integrated in a poly (vinyl alcohol) (PVA) matrix. These sensors can achieve oxygen sensing in a reversible manner, quenching the fluorescence intensity in the range of 0–8 kPa O_2_.

Despite the complexity of the reaction pathways during the hydrothermal synthesis, we have identified by electrospray ionization coupled with quadrupole time of flight (ESI-QTOF) and liquid chromatography coupled with mass spectrometry (LC-MS) Bandrowski’s base as the main component at 16 h reaction times.

To the best of our knowledge, this work represents the first example of a multi-emissive oxygen probe with a fluorescence-based oxygen-quenching mechanism. This work has the potential to set an industrially-viable approach for the development of purely organic fluorescence-based reversible oxygen sensors.

## 2. Materials and Methods

### 2.1. Materials

PPD (≥99%), and urea (≥99%) were obtained from Sigma-Aldrich (St. Louis, MO, USA) for the synthesis of the fluorophores. Potassium Bromide (KBr) of analytical grade (≥99%) was purchased from Merck (Darmstadt, Germany) for the FT-IR studies. HPLC graded acetonitrile (ACN) (Sigma-Aldrich, ≥99.9%) and ultra-pure water (milli-Q) has been used as eluent for both LC-MS and flash chromatography. Mowiol 40–88 (M_w_ ~ 205,000) was purchased from Sigma-Aldrich for the embedment of the optical sensor. Oxygen (99.99% purity, H_2_O < 2 ppm) and nitrogen (99.8%, H_2_O < 40 ppm) tanks were purchased from Carbagas.

### 2.2. Synthesis of the Fluorophores

A quantity of 100 mg of PPD and 100 mg of urea were placed in a round-bottom flask in the presence of 25 mL of water. Subsequently, the solution was vigorously mixed by sonicating for 30 min at RT, either in air or nitrogen atmosphere. Afterward, the solution was transferred to a polytetrafluoroethylene (PTFE) lined synthesis autoclave reactor (Parr Instrument, Acid Digestion Vessel) with a volume of 50 mL. For the de-oxygenated conditions, the solution was degassed by bubbling nitrogen under sonication and the Teflon chamber was flushed with nitrogen before transferring the solution. The PTFE lined autoclave reactor was then placed in an oven and maintained at 160 °C for different reaction times (Table 1). The products were then freeze-dried for 24 h. Accordingly, the products are named as U-PPDx, Uy, PPDz, D-Um, D-PPDn, and D-U-PPDo respectively, where x, y, z, m, n, and o represent the reaction time, U-PPD represents the used reactants for the synthesis (U: urea and PPD: p-phenylenediamine. To synthesize U16 and PPD16, we have utilized the same procedure but with a concentration of the single precursor equal to 8 mg/mL.

### 2.3. Apparatus and Methods

Molecular weight analysis of U-PPD16 and its isolated compounds were performed with ESI-QTOF, MALDI-TOF and LC-MS. A mid-mass positive method ESI-QTOF was combined with mass spectroscopy for U-PPD2, U-PPD4, U-PPD6, U-PPD8 and U-PPD16 samples within the range from 100 to 1000 *m*/*z*. For MALDI-TOF experiments, MALDI-TOF UltraFlex LP 5–20 kDa using a 10/1 trans-2-[3-(4-tert-Butylphenyl)-2-methyl-2-propenylidene] malononitrile (DCTB): Na matrix for U-PPD2 and U-PPD8 samples. LC-MS measurements, a mid-mass polar method coupled with UV–Vis detector was chosen to study the mass separation ranging between 200–1000 *m*/*z* for U-PPD2, U-PPD8, U-PPD16 and isolated compounds of U-PPD16.

Compounds corresponding to different emission centers were isolated from multi-emissive fluorophores by flash chromatography using a three-way pump (Buchi Sepacore, C-601, Buchi AG, Flawil, Switzerland) connected to a pump controller module (Buchi, C-610, Buchi AG, Flawil, Switzerland) coupled to an Ultrapure Silica Reverse Phase column (Buchi Cartridge, RP18ec, Buchi AG, Flawil, Switzerland). 500 mL solutions of ACN/water in different percentages (5%, 15%, 30%, 50%, 90% and 100%) were used as mobile phase/eluent. A sample containing 4% *w*/*v* U-PPD16 in 50/50 ACN: water was injected by a valve into the reverse phase column.

FT-IR spectrum were recorded with Varian-640-IR (Portmann Instruments GmbH, Biel-Benken, Switzerland). The conventional KBr pellet procedure (0.05% *w*/*w* of samples in KBr) was used for all samples. The FT-IR absorbance spectrum was recorded at frequency range of 400–4000 cm^−1^ with 4 cm^−1^ resolution.

The chemical composition of the fluorophores was characterized using X-ray photoelectron spectroscopy (XPS, PHI VersaProbe II, Physical Electronics, Chanhassen, MN, USA). The energy resolution and pass energy were 0.8 eV/step and 187.85 eV for survey scans and 0.125 eV/step and 29.35 eV for high-resolution scans, respectively. Carbon 1 s at 284.5 eV was used as a reference to correct for charge effects. Samples were pressed onto Indium foil (Alfa Aesar, 99.99% purity, Kandel, Germany).

AFM images were obtained with Nanosurf Ambient AFM variant (Flex-AFM, Nanosurf AG, Liestal, Switzerland) by utilizing AFM probe of SHR150 (BudgetSensors, Sofia, Bulgaria) with spring constants of 5 N/m and resonant frequency of 150 kHz. Aqueous solutions of U-PPD2, U-PPD4, U-PPD6, U-PPD8 and U-PPD16 with con-centration of 0.005% *w*/*v* and volume of 50 µL were drop-cast onto mica sheets (Science Services, 15 × 15 mm and 0.2 mm thickness, Munich, Germany) and completely dried for 2 h at room temperature before starting the AFM measurement.

The UV–Vis absorption spectra for all samples were measured at room temperature with SynergyMx, Biotek Instruments GmbH (Bad-Friedrichshall, Germany) in the wavelength range of 250–800 nm with 2 nm increments.

Fluorescence measurements were performed by Horiba Jobin Yvon FluoroMax-4 (Horiba Jobin Yvon GmbH, Bensheim, Germany) for all samples. A concentration of 0.005% *w*/*v* was utilized for all samples since it gives the highest fluorescence emission. A range of excitation and emission wavelengths of 300–600 nm and 300–800 nm were used, respectively. A slit width of 5 nm was used for all samples for both excitation and emission wavelengths. Lifetime measurements for all samples were performed by Horiba DeltaPro (Horiba Jobin Yvon GmbH, Bensheim, Germany) for U-PPD2 and U-PPD16 at excitation wavelengths of 366 nm. LUDOX solution was used to calculate the instrument response factor (IRF). The quantum yield of U-PPD16 was measured directly with a spectrofluorometer (JASCO FP-8500, JASCO Inc., Easton, MD, USA) equipped with a 100 mm-diameter integrating sphere.

### 2.4. Preparation of Polyvinyl Alcohol (PVA)/U-PPD16 Film

A solution of 0.005% *w*/*v* of U-PPD16 containing 5% *w*/*v* Mowiol 40–88 in water was prepared and vigorously stirred at 60 °C for 12 h. 1.5 mL of this solution was drop-casted on a polystyrene petri dish (d = 14 cm) and dried at 50 °C for 24 h to let the water evaporate. The resulting films had a thickness of 200 ± 20 μm.

### 2.5. Oxygen Response Measurement of Polyvinyl Alcohol (PVA)/U-PPD16 Film

To measure the oxygen response, oxygen/nitrogen ratios of 0%, 1%, 2%, 4%, 8% and 21% were purged within the spectrofluorometer chamber by mixing pure N_2_ and O_2_ gas with a flow-mixer setup (OxiQuant S, Envitec GmbH, Wismar, Germany). Before each measurement, the sample was equilibrated at 100% N_2_ for 30 min before it was purged with the targeted O_2_/N_2_ ratio for another 30 min.

Fluorescence measurements of U-PPD16 embedded in PVA films were performed within excitation wavelengths and emission wavelengths of 300–600 nm and 300–800 nm, respectively. A slit width of 5 nm was used for all samples for both excitation and emission wavelengths.

## 3. Results

### 3.1. Chemical Characterization of the Multi-Emissive Optical Probes

**Figure 1:**
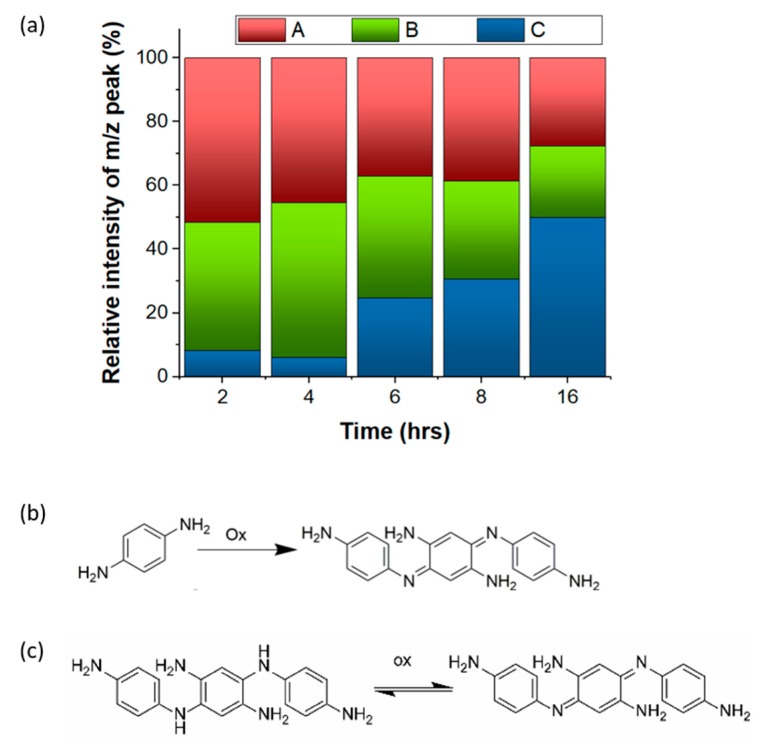
(**a**) Relative chemical composition (%) of products obtained during the hydrothermal synthesis with urea and PPD at different reaction times as determined by ESI-QTOF, namely compound A (560.985 *m*/*z*), B (426.946 *m*/*z*) and C (319.166 *m*/*z*) (**b**) Oxidative oligomerization of PPD to Bandrowski’s base, (**c**) different oxidative states of Bandrowski’s base.

To investigate the mass distribution and relative concentrations of the individual fluorophores in the hydrothermal products synthesized with urea and PPD, we have used multiple mass spectroscopic techniques, namely MALDI-TOF, ESI-QTOF, and LC-MS. MALDI-TOF of U-PPD2 and U-PPD8 (Figure S1a,b) did not show any compound with *m*/*z* higher than 1000. This size corresponds to globular particles with a diameter of approximately 0.66 nm [30]. This is in contradiction with a previous study showing the formation of nanostructures (average size of 2.6 nm as calculated by high resolution transmission electron microscopy (HR-TEM)) with the same chemistry [31]. However, in our case, we could demonstrate by AFM imaging that the particles disappeared at higher reaction times (t = 16 h) (Figure S2). At the same time, three main reaction products indicated by ESI-QTOF in the lower molecular weight region (100–1000 *m*/*z*) (Figure 1a), namely compound A (560.985 *m*/*z*), B (426.946 *m*/*z*) and C (319.166 *m*/*z*), persist during the entire analyzed time range, although their relative intensity varied. This indicates that only low molecular weight fluorophores are responsible for the measured fluorescence emission.

If the reaction time is increased above 8 h, the compound C (319.166 *m*/*z*) constitutes the main product. This mass is consistent with the molecular weight of Bandrowski’s base, a trimeric stable PPD oligomer produced by the self-polymerization of PPD with oxidizing agents under alkaline conditions (Figure 1b) [32]. Indeed, the relative intensity of the peak attributed to Bandrowski’s base was only ~8% for U-PPD2 whereas it reached up to 50% for U-PPD16 (Figure 1a). The presence of Bandrowski’s base as the main product at higher reaction times was also confirmed with LC-MS (Table S1a,b). Combining these findings, we assume that the formation of the Bandrowski’s base predominantly occurs as the reaction proceeds.

**Figure 2:**
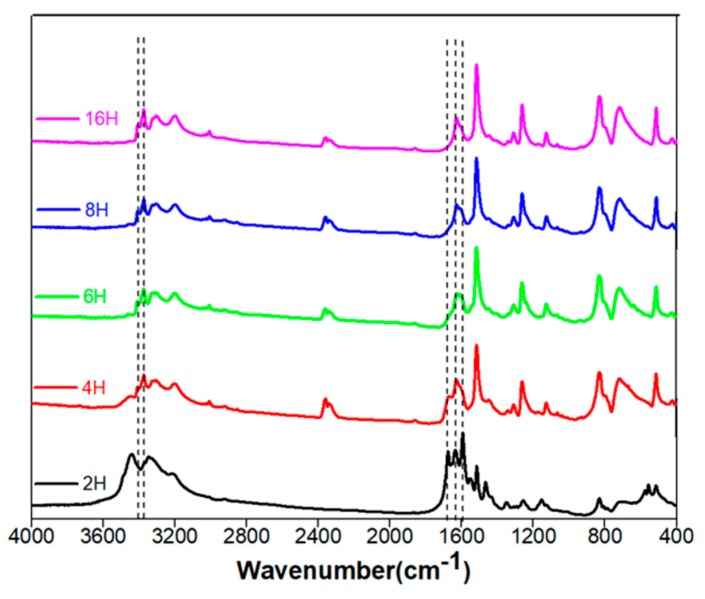
FT-IR spectra of U-PPD at 2, 4, 6, 8 and 16 h reaction time.

To understand which other groups of molecules are synthesized at shorter reaction times and partially retained even after 16 h, we performed FT-IR on the U-PPD samples from 2 to 16 h (Figure 2). FT-IR measurements showed that at reaction times between 2 and 4 h, the symmetric and asymmetric stretching (C=O)NH vibrations (amide I) of the urea bond [33] at 1594 cm^−1^ and 1635 cm^−1^ almost disappeared. Simultaneously, the intensities of the peak corresponding to C=O stretching vibrations of the carbonyl group of urea at 1675 cm^−1^ decreased as the reaction proceeded. By contrast, peaks corresponding to symmetric and asymmetric NH_2_ stretching vibrations of primary amines of PPD located at 3328 and 3372 cm^−1^ [34] increased in intensity as the reaction proceeded. A simultaneous decrease of urea functional units and an increase of amine units may suggest that polyaromatic ureas were synthesized at shorter reaction times (U-PPD2), then converted back to PPD (U-PPD4, U-PPD6 and U-PPD8), which subsequently formed oligomers of aromatic di-amines at longer reaction times (U-PPD16). This finding is also in agreement with previously reported reaction pathways involved in high-temperature reactions of the single components or their mixtures [35].

To get information on elemental composition and relative abundancy of functional groups for each hydrothermal product, we performed XPS on the U-PPD samples from 2 h to 16 h. XPS high-resolution region scans confirmed the set of structures of the fluorescent products suggested by ESI-QTOF, namely urea precursors, polyaromatic urea units, unreacted and oligomerized PPD. Four different C1s signals located at 284.5 eV, 285.5 eV, 286.6 eV, and 288.5 eV were fitted and ascribed to C-C/C-H, C-N, C=N, and N-C=O in agreement with previous literature [36,37,38] (Figure S4). The relative atomic concentration of carbon (C1s) remained constant (~71%) over the entire synthesis whereas nitrogen (N1s) concentration decreased by 7% as the reaction proceeded from 2 to 16 h, suggesting the elimination of nitrogen-rich volatile products, such as ammonia (Table S2). This may indicate the release of nitrogen-rich volatile products as a consequence of the decomposition of polyaromatic urea units.

**Figure 3:**
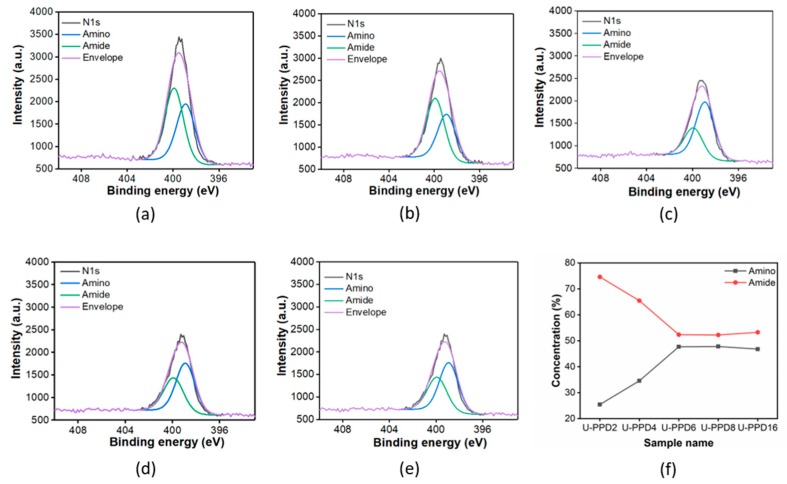
High resolution XPS spectra of N1s for U-PPD2 (**a**), U-PPD4 (**b**), U-PPD6 (**c**), U-PPD8 (**d**) and U-PPD16 (**e**). XPS analysis of U-PPD samples from 2 h to 16 h indicating the amino (black) and amide (red) group change with respect to the reaction time (**f**).

In addition, the N1s peak can be fitted with two main peaks, ascribed to amino (-NH_2_) (398.9 eV) and amide (-CONH_2_/-CONHR/-CONR) (399.9 eV) functionalities [39,40] (Figure 3a–e). Interestingly, by monitoring their relative concentration change as a function of time, we could observe that the amide bond ratio among N-rich moieties is decreasing up to a reaction time of 6 h and stabilizes thereafter (Figure 3f). This also confirms that the predominant urea-bonds formed between aromatic moieties are partly decomposed, resulting in PPD monomers that can subsequently form PPD trimers, thereby displaying different oxidative states for the nitrogen (Figure 1c).

### 3.2. Optical Characterization of the Multi-Emissive Pattern

**Figure 4:**
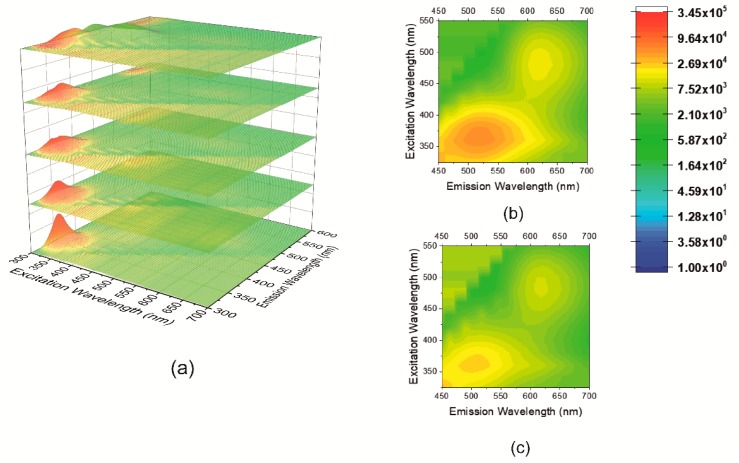
(**a**) 2D fluorescence surface color map of kinetic samples for U-PPD at reaction times of 2, 4, 6, 8 and 16 h (from bottom to top), 2D contour color map of U-PPD16 (**b**) and U-PPD8 (**c**) highlighting the third (λ_exc3_ = 360 nm, λ_em3_ = 516 nm) and the fourth (λ_exc4_ = 480 nm, λ_em4_ = 618 nm) emission center.

To identify the effect of the chemical nature of fluorophores on optical properties, we monitored the evolution of each emission center by recording multi-emission fluorescence patterns for U-PPD at reaction times of 2, 4, 6, 8, and 16 h. Four emissive states were measured at a reaction time of 16 h (λ_exc1_ = 303 nm, λ_em1_ = 358 nm, λ_exc2_ = 320 nm, λ_em2_ = 390 nm, λ_exc3_ = 360 nm, λ_em3_ = 516 nm, λ_exc4_ = 480 nm, λ_em4_ = 618 nm) (Figure 4a). A quantum yield of 25.1% was determined for U-PPD16, which is consistent with literature where 24% was reported [31].

Bandrowski’s base has been identified as the major component at a reaction time of 16 h (Figure 1a). Therefore, this compound was synthesized according to a previous protocol and we recorded its fluorescence spectrum [41] (λ_exc_ = 580 nm, λ_em_ = 610 nm) (Figure S7). While the fluorescence emission of U-PPD16′s fourth emission center is similar to that of Bandrowski’s base, its excitation is blue-shifted by 100 nm in the present hydrothermal reaction (Figure 4b,c). This increase in optical bandgap might be related to energy transfer processes that occur with the other fluorophores present in the reaction medium.

To investigate the chemical structures of the fluorophores corresponding to the other three emission centers (λ_exc1_ = 303 nm, λ_em1_ = 358 nm, λ_exc2_ = 320 nm, λ_em2_ = 390 nm, λ_exc3_ = 360 nm, λ_em3_ = 516 nm), we characterized the fluorescence emission of hydrothermal products from the individual precursors as well as from the mixture. The spectra were recorded at a reaction time of 16 h by utilizing air or oxygen-free headspace in the hydrothermal vessel (Figure S5). For pure D-PPD16, we observed an excitation peak at λ_exc_ = 360 nm and an emission peak at λ_em_ = 520 nm (Figure S5a). The emission peak was red-shifted if the reaction atmosphere was standard air (PPD16 (λ_exc_ = 360 nm and λ_em_ = 540 nm)) (Figure S5b). This may indicate that oxidative oligomerization of PPD is predominantly occurring under standard air conditions, resulting in red-shifting of the emission peak due to the increased π conjugation. The emission pattern of D-PPD16 and PPD16 strongly resembles the third emission peak of U-PPD16 located at λ_exc3_ = 360 nm, λ_em3_ = 516 nm, which suggests that oligomeric forms of PPD might be responsible for this emission center.

The simultaneous presence of two emissive states within different oligomeric forms of PPD can be related to the occurrence of a redox equilibrium between PPD and benzoquinone di-amines moieties (Figure 1c). This is further confirmed by the increased fluorescence intensity at λ_em3_ = 516 nm. Compared to D-U-PPD16, U-PPD16 showed an increase of intensity by 76% (Figure S5c,d). Absorption studies further confirmed the presence of both benzenoid rings of the oligomeric derivatives of PPD [42] with π–π* transitions at 292 nm and 312 nm and quinoid rings in *p*-phenylene quinone diimine (PPQD) [43] with π–π* transition at 415 nm. At higher reaction times, oligomerization of PPQD is observed by the occurrence of a broader adsorption located at 485 nm (t = 16 h). Indeed, this absorption band at 485 nm is consistent with the absorption of Bandrowski’s base (i.e., 480 nm at a pH value of 7.5) [44] (Figure S3). This finding is also consistent with the 2D contour color maps of U-PPD16 and U-PPD8 (Figure 4b,c) showing the evolution of the fourth emission center (λ_exc4_ = 480 nm, λ_em4_ = 618 nm) as the reaction proceeds.

The fluorescence intensity ratio between the second emission center (λ_exc2_ = 320 nm, λ_em2_ = 390 nm), which appears as early as U-PPD4, and the first emission center (λ_exc1_ = 303 nm, λ_em1_ = 358 nm) strongly increases at longer reaction times, as indicated in the 2D fluorescence contour plots (Figure 4). This may suggest that PPD is slowly converted back from aromatic polyurea compounds. Indeed, the PPD precursor (p-phenylenediamine) emission is located at 385 nm (Figure S6). If this hypothesis is valid, we can assign the first emission center (λ_exc1_ = 303 nm, λ_em1_ = 358 nm) to aromatic polyurea compounds. Despite their higher conjugation, the relatively high optical bandgap might be due to multiple orbital nodes among their structures.

The lifetime decay curve for U-PPD2 at an excitation wavelength of 366 nm was bi-exponential (τ_1_ = 0.43 ns, τ_2_ = 3.27 ns; τ_average_ = 0.58 ns). Interestingly, we observed an additional component with longer lifetime for U-PPD16. The average lifetime of U-PPD16 increased approximately six-fold (τ_1_ = 0.55 ns, τ_2_ = 3.21 ns, τ_3_ = 6.15 ns; τ_average_ = 3.01 ns) (Table S3). This could be due to the formation of new oligomeric structures of PPD and its derivatives at increasing reaction times.

The excitation-dependent emission pattern observed for all emission centers is likely a result of the broad structural distribution of PPD-derivatives, similar to what has been simulated with low molecular weight polycyclic aromatic moieties for citric acid-based CNDs [45]. Isolation of the three emission centers by flash chromatography further confirmed the different chemical nature of the fluorophore mixture. The emission center related to oligomeric forms of PPD located at 511 nm was extracted by collecting the fractions with 5% *v*/*v* of ACN using water as eluent. The other two emission centers related to the single PPD precursor (λ_em2_ = 402 nm) and Bandrowski’s base (λ_em2_ = 606 nm) nm were eluted with 15% *v*/*v* of CAN in water (Figure S8).

### 3.3. Fluorescent PVA Films and Their Multi-Emissive Oxygen Sensing Properties

The previously reported solvothermal synthesis of CNDs from m-phenylenediamine [26], ethylenediaminetetracetic acid [16] and isophorone di-isocyanate [17] resulted in a set of multi-emissive fluorophores. To test if this is also the case if the PPDs synthesized here displays RTP emission when embedded into a PVA matrix (Mowiol 40–88), we measured the luminescence emission before and after encapsulation within the matrix.

The emission center previously located at λ_exc2_ = 320 nm, λ_em2_ = 390 nm, λ_exc3_ = 360 nm, λ_em3_ = 516 nm, λ_exc4_ = 480 nm, λ_em4_ = 618 nm (Figure 4a) underwent blue-shifts in the emission by 80 nm, 100 nm and 60 nm, respectively. Meanwhile, their intensity increased by 21%, 56% and 32%, respectively (Figure S9 vs. Figure 4a). Similarly, an increased intensity has been observed for CNDs synthesized from ethanediamine precursors if they were embedded into PVA films and fibers [46]. A blue-shift has been observed in a similar system where CNDs synthesized from *m*-phenylenediamine were embedded into a PVA matrix, and the blue shift was ascribed to the absence of any solvent relaxation [26].

**Figure 5:**
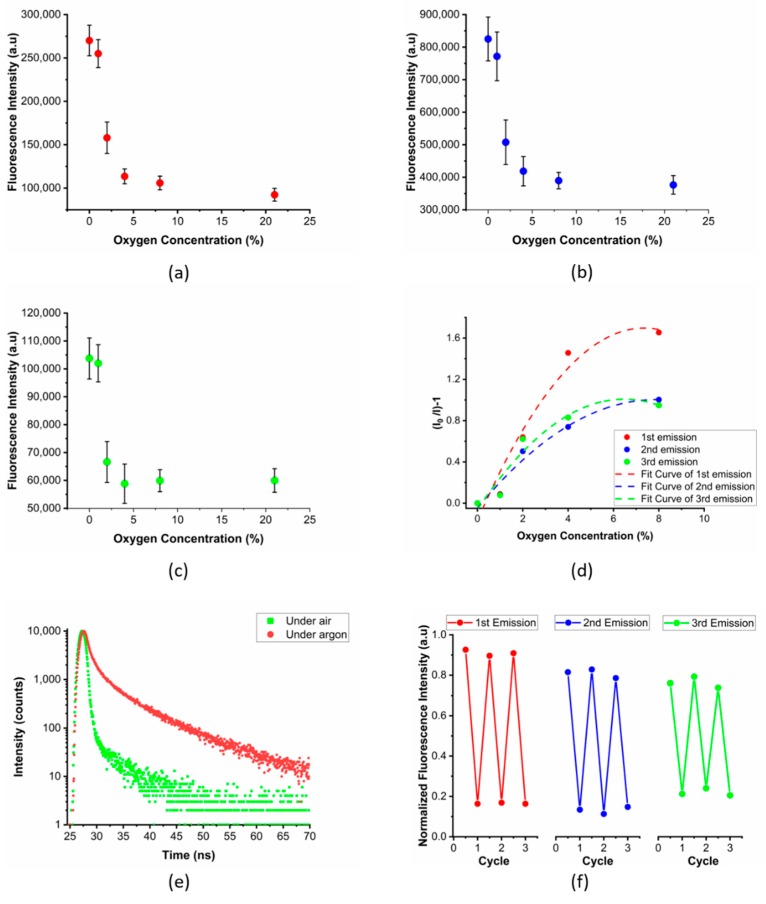
Fluorescence intensity of polymer films composed of U-PPD16 embedded in a PVA matrix (Mowiol 40–88) as a function of the oxygen concentrations for the emission centers of 310 nm (**a**), 400 nm (**b**) and 550 nm (**c**). (**d**) Non-linear Stern–Volmer calibration plot of each emission center and 2nd order polynomial fit. (**e**) Fluorescence emission decay curve of the functional PVA film under air and argon. (**f**) Reversibility of the functional PVA film for each emission center during three consecutive 0–21 kPa O_2_ cycles.

To investigate the possible oxygen sensitivity of these multi-emissive fluorescent PVA films, we recorded their 2D fluorescence contour plots at 0, 1, 2, 4, 8 and 21 kPa O_2_ (Figure 5a–c). Fluorescence quenching was observed for all three emission centers when increasing the O_2_/N_2_ ratio up to 8 kPa of O_2_. The resultant Stern-Volmer plots (Figure 5d) were fitted to the second-order polynomial function (R^2^ equal to 0.964, 0.928 and 0.941 for 1st, 2nd and 3rd emission center, respectively). The limit of detection (LOD) was calculated according to the extrapolated concentration at which the signal is three times the averaged standard deviation (3σ) of the fluorescence intensity. The LOD of each emission center was 0.886, 0.669 and 0.755 kPa, respectively. The fitting showed a non-linear calibration plot which confirms that the sensor microenvironment plays a role in the collisional quenching between the luminescent dye and oxygen [47]. To exclude any irreversible photo-oxidation processes, we performed up to three 0–21 kPa O_2_ cycles (Figure 5f). The excellent reversibility suggests that fluorescence quenching is provoked by the electron transfer to the excited oxygen singlet state.

To test the sensor cross-interference to ambient relative humidity and CO_2_, we recorded the 2D fluorescence contour plots of fluorescent PVA films under ambient air and O_2_/N_2_ dry gas mixture conditions. The emission wavelength and intensity did not change for all three emission centers when the fluorescent PVA films were exposed to ambient air conditions and subsequently purged with O_2_/N_2_ dry gas mixture at an oxygen pressure of 21 kPa (Figure S10). The lack of interactions between the emission centers and water/CO_2_ was confirmed by the retention of the fluorescent features.

To investigate the quenching mechanism, we also performed lifetime measurements under air and argon atmosphere, upon excitation at 366 nm. The average lifetime of fluorescent PVA films (τ_average_ = 6.6 ns in argon and τ_average_ = 0.4 ns in air) was shorter compared to that of phosphorescence (from μs to s) (Figure 5e). This indeed confirms that quenching of fluorescence is triggered by oxygen although no transition to the triplet state is observed. While the exact nature of this mechanism is still under debate, a similar behavior has already been observed in other N-rich aromatic rings such as β-carbolines [27] and acridines [28]. This is reputed to occur as a result of the formation of charge transfer complexes between the contact radical ion pair state ^3^(^2^M^•+^,2O^2•−^) when mixed with the locally excited (LE) states [29]. Possibly these intermediate energy level states enable the energy matching with the triplet ground state and excited singlet state bandgap of molecular oxygen.

Although the precise underlying mechanism remains unclear, we demonstrate the first example of multi-emissive optical oxygen sensing. Such a feature is particularly advantageous for the reversible and non-destructive monitoring of oxygen. Future studies will verge on understanding the exact role of aromatic amines in oxygen quenching once they are embedded within a hydrogen bonding matrix.

## 4. Conclusions

Multi-emissive fluorophores were produced through a hydrothermal synthesis with urea and PPD as starting materials. From ESI-QTOF analysis, we identified three main components whose ratios vary as a function of the reaction time. At longer reaction times (t >8 h), Bandrowski’s base, a trimer of PPD, constitutes the main component. Once U-PPD16 is embedded in a PVA matrix, we demonstrated that the films undergo oxygen-induced fluorescence quenching in all three emission centers. The quenching follows the non-linear Stern–Volmer plots and gives a LOD of 0.886, 0.669 and 0.755 kPa for each emission center, respectively. Moreover, the oxygen sensor showed reversibility during up to three cycles, which could pave the way for the design of multi-emissive optical oxygen sensors. Thanks to the cost-effective and metal-free formulation, these sensors have the potential to be used in food packaging to monitor the oxygen concentration by cross-referencing each single emission center to the relevant dynamic oxygen range. In addition, this work opens up new perspectives in the development of fluorescence-based optical oxygen sensors.

## Figures and Tables

**Table 1:** Hydrothermal reaction condition for producing urea and PPD derived fluorophores. 100 mg urea and 100 mg PPD in 25 mL water were placed in an autoclave reactor. The reaction time was kept between 2 h and 16 h. The final mass was divided by the initial total mass of precursors to calculate the percentage of obtained mass. ^a^ Not determined.

Sample Name	Reaction Atmosphere	Reaction Time (h)	Precursors	Final/Initial Mass (%)
U-PPD2	Air	2	Urea-PPD	71.2
U-PPD4	Air	4	Urea-PPD	56.6
U-PPD6	Air	6	Urea-PPD	53.8
U-PPD8	Air	8	Urea-PPD	56.8
U-PPD16	Air	16	Urea-PPD	57.5
U16	Air	16	Urea	32.7
PPD16	Air	16	PPD	51.8
D-U16	Nitrogen	16	Urea	N.D.^a^
D-PPD16	Nitrogen	16	PPD	N.D.^a^
D-U-PPD16	Nitrogen	16	Urea-PPD	N.D.^a^

## Supplementary Materials

The following are available online at https://www.mdpi.com/1424-8220/20/2/477/s1. Table S1. LC-MS peak analysis of U-PPD2 (a) and U-PPD8 (b) with the molecular weight, retention time, and area % total. Table S2. Elemental composition of U-PPD2, U-PPD4, U-PPD6, U-PPD8 and U-PPD16 by XPS. Table S3. Lifetime measurements of U-PPD2 (a) and U-PPD16 (b). Figure S1. MALDI-TOF analysis of U-PPD2 (a) and U-PPD8 (b), ESI-QTOF analysis of U-PPD2 (c), U-PPD4 (d), U-PPD6 (e), U-PPD8 (f) and U-PPD16 (g). Reference *m*/*z* values for ESI-QTOF analysis for all kinetic samples (h). Figure S2. Dynamic mode AFM images of U-PPD2 (a), U-PPD6 (b), U-PPD8 (c) and U-PPD16 (d). Figure S3. UV-Vis spectra of U-PPD at 2, 4, 6, 8 and 16 hrs reaction time. Figure S4. High resolution XPS spectra of C1s for U-PPD2 (a), U-PPD4 (b), U-PPD6 (c), U-PPD8 (d) and U-PPD16 (e). Figure S5. 2D fluorescence color map of PPD16 (a), D-PPD16 (b), U-PPD16 (c) and D-U-PPD16 (d). Figure S6. 2D fluorescence contour plot of p-phenylenediamine. Figure S7. 2D fluorescence color map of Bandrowski's Base synthesized according to a previous literature method. Figure S8. 2D fluorescence color map of isolated compounds with emission centers located at λ_exc_= 340 nm, λ_em_ = 402 nm (a), λ_exc_ = 420 nm, λ_em_ = 511 nm (b) and λ_exc_ = 520 nm, λ_em_ = 606 nm (c). Figure S9. 2D fluorescence contour plot of U-PPD16 embedded into Mowiol 40-88 (PVA). Figure S10. 2D flourescence contour plot of U-PPD16 embedded into Mowiol 40-88 under ambient condition (a) and 21 kPa O_2_ (b). Figure S11. Response time of the third emission center of U-PPD16 embedded into Mowiol 40-88.
