# Supplementary material for: Reversible Oxygen Sensing Based on Multi-Emission Fluorescence Quenching
Source: Sensors (Basel). 2020 Jan 15;20(2):477. doi: 10.3390/s20020477 (PMC7014081; doi:10.3390/s20020477)
Supplement: Supplementary file 1 [file sensors-20-00477-s001.pdf]

sensors

Supporting Information

Reversible Oxygen Sensing Based on Multi-Emission Fluorescence Quenching

Table S1. LC-MS peak analysis of U-PPD2 (**a**) and U-PPD8 (**b**) with the molecular weight, retention time, and area % total.

Peak Number	Dominant Molecular Weight (Da)	Retention Time (min)	Area % Total
#1	319.3	1.49	1.13
#2	138.9	1.94	11.14
#3	138.9	2.05	19.98
#4	213.1	2.22	10.69
#5	213.1	2.31	19.17
#6	Multiple	2.45	6.82
#7	586.5	2.53	6.45
#8	543.5	2.71	14.59
#9	Multiple	4.79	8.39
#10	131.6	5.94	1.62

(**a**)

Peak Number	Dominant Molecular Weight (Da)	Retention Time (min)	Area % Total
#1	Multiple	0.57	7.73
#2	319.3	1.65	39.21
#3	214.1	1.81	2.54
#4	410.3	2.10	5.83
#5	410.3	2.15	6.51
#6	205.2	2.19	10.38
#7	408.1	2.27	3.00
#8	213.2	2.41	14.99
#9	340.5	2.52	5.48
#10	340.5	2.75	4.32

(**b**)

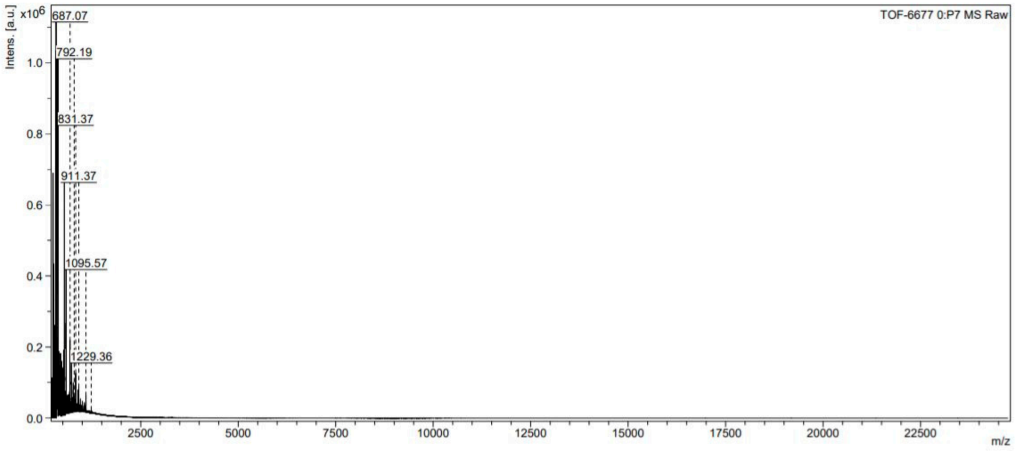

(a)

Sensors **2020**, *20*, 477; doi:10.3390/s20020477 www.mdpi.com/journal/sensors

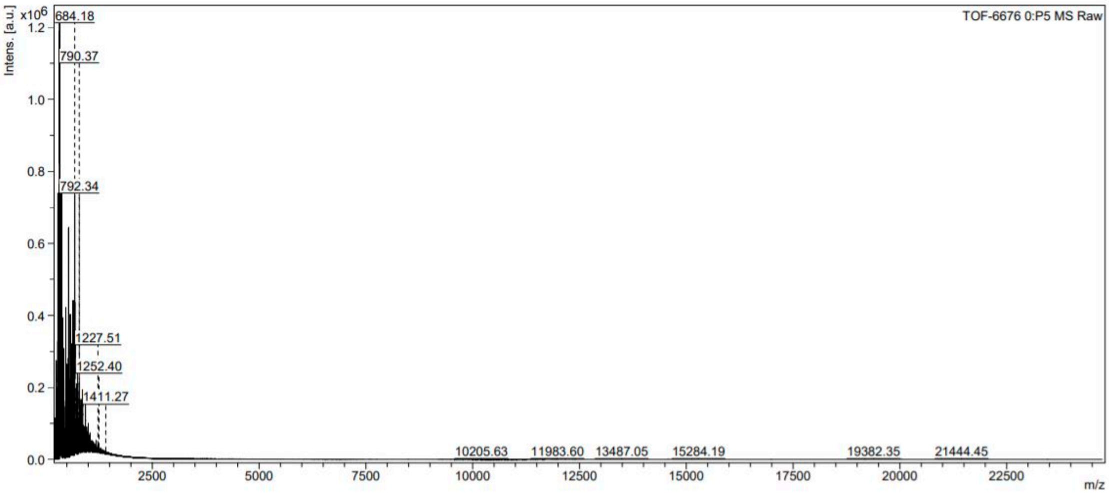
Intens. [a.u.]
x10^6
TOF-6676 0:P5 MS Raw
684.18
790.37
792.34
1227.51
1252.40
1411.27
10205.63
11983.60
13487.05
15284.19
19382.35
21444.45
m/z

(**b**)

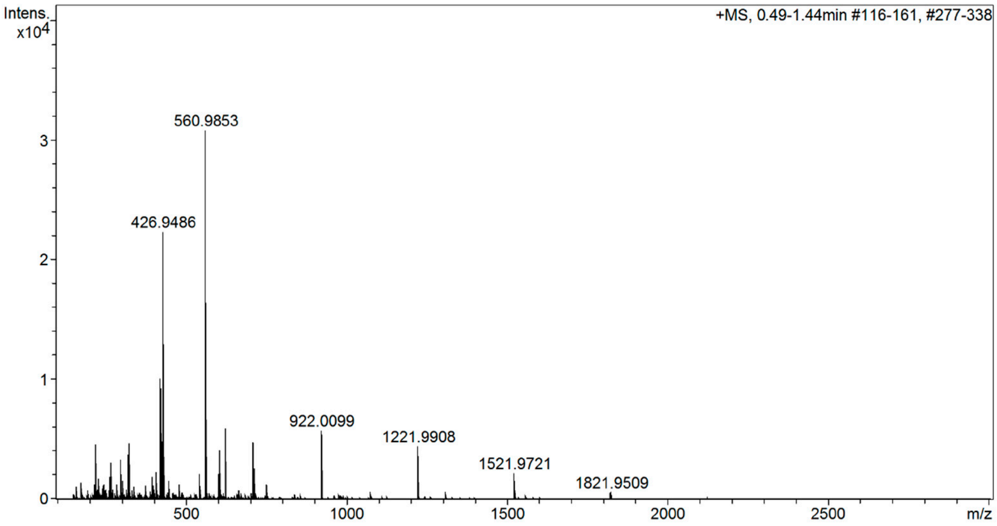
Intens.
x10^4
+MS, 0.49-1.44min #116-161, #277-338
560.9853
426.9486
922.0099
1221.9908
1521.9721
1821.9509
m/z

(**c**)

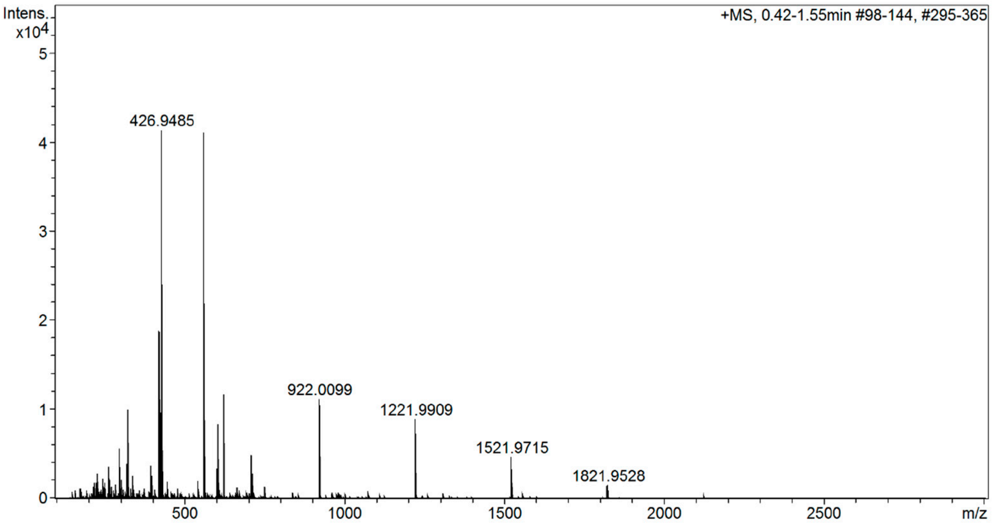
Intens.
x10^4
+MS, 0.42-1.55min #98-144, #295-365
426.9485
922.0099
1221.9909
1521.9715
1821.9528
m/z

(**d**)

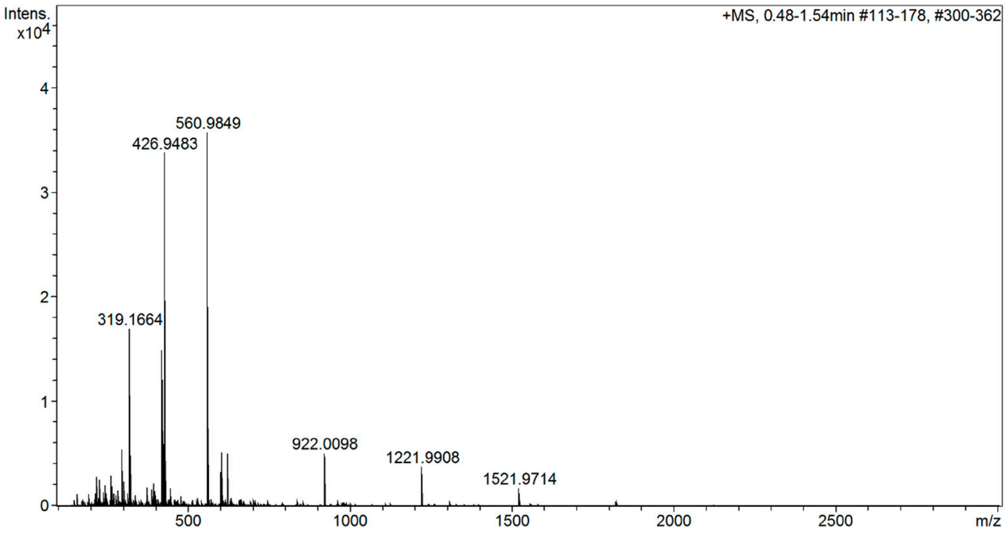
Intens.
x10^4
+MS, 0.48-1.54min #113-178, #300-362
560.9849
426.9483
319.1664
922.0098
1221.9908
1521.9714
m/z

(**e**)

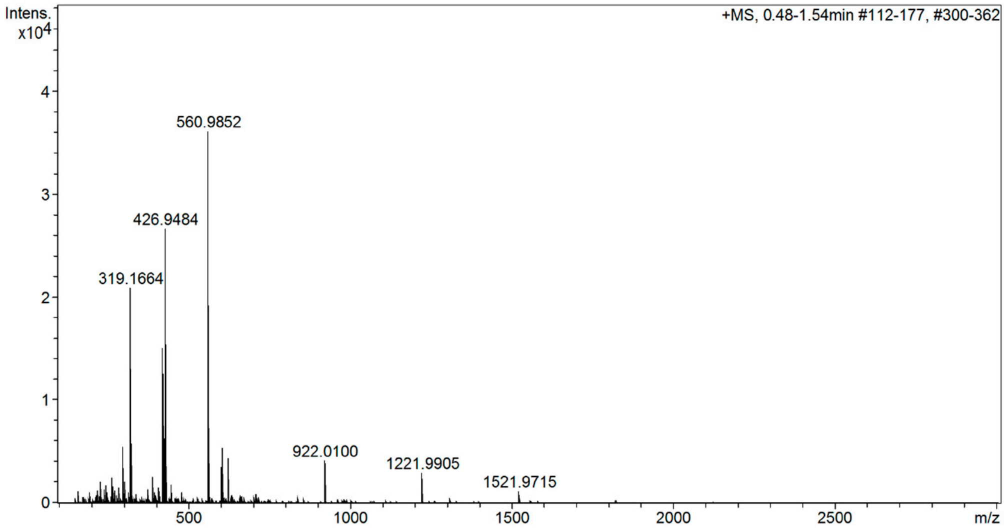
Intens.
x10^4
+MS, 0.48-1.54min #112-177, #300-362
560.9852
426.9484
319.1664
922.0100
1221.9905
1521.9715
m/z

(**f**)

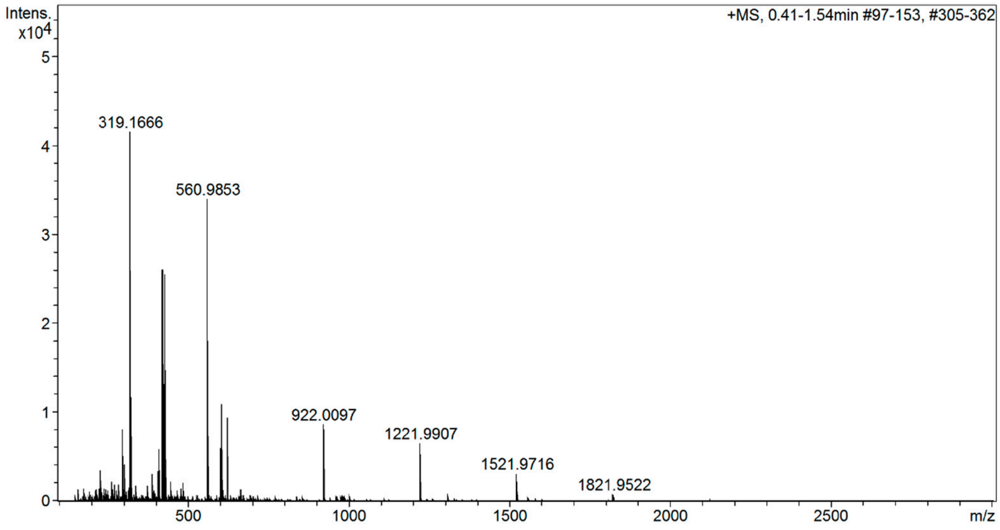
Intens.
x10^4
+MS, 0.41-1.54min #97-153, #305-362
319.1666
560.9853
922.0097
1221.9907
1521.9716
1821.9522
m/z

(**g**)

Reference m/z	Resulting m/z	Intensity	Error [ppm]
118.0863			
322.0481	322.0481	4658	0.070
622.0290	622.0288	5908	-0.226
922.0098	922.0099	5753	0.069
1221.9906	1221.9908	4455	0.143
1521.9715	1521.9721	2176	0.400
1821.9523	1821.9509	563	-0.796
2121.9332	2121.9339	199	0.340
2421.9140			
2721.8948			

Standard deviation: 0.599

(**h**)

Figure S1. MALDI-TOF analysis of U-PPD2 (**a**) and U-PPD8 (**b**), ESI-QTOF analysis of U-PPD2 (**c**), U-PPD4 (**d**), U-PPD6 (**e**), U-PPD8 (**f**) and U-PPD16 (**g**). Reference m/z values for ESI-QTOF analysis for all kinetic samples (**h**).

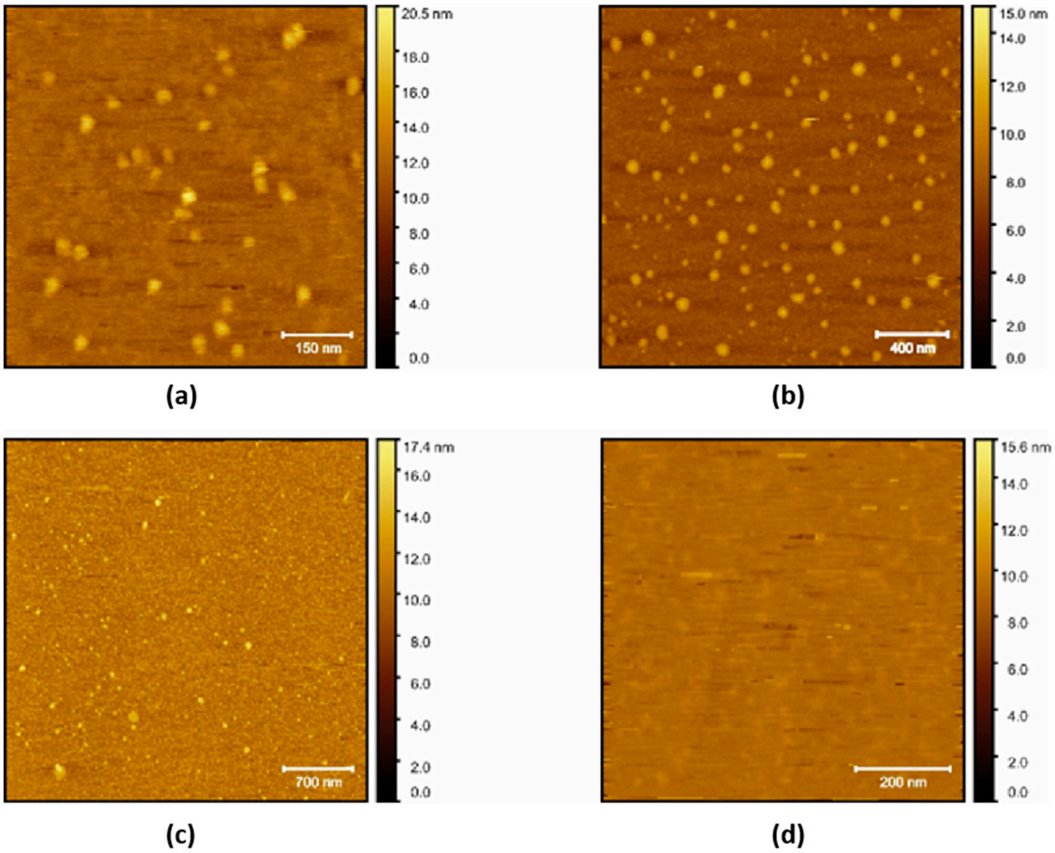

Figure S2. Dynamic mode AFM images of U-PPD2 (**a**), U-PPD6 (**b**), U-PPD8 (**c**) and U-PPD16 (**d**).

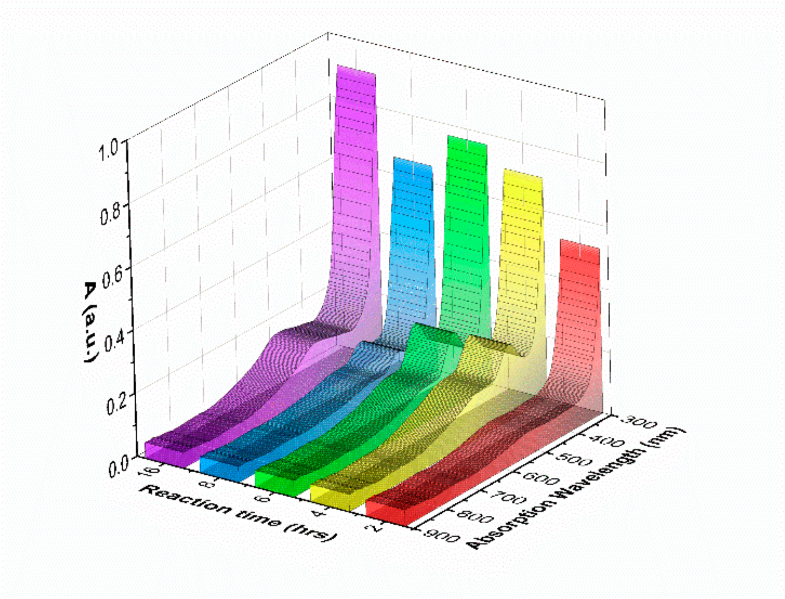

Figure S3. UV-Vis spectra of U-PPD at 2, 4, 6, 8 and 16 hrs reaction time.

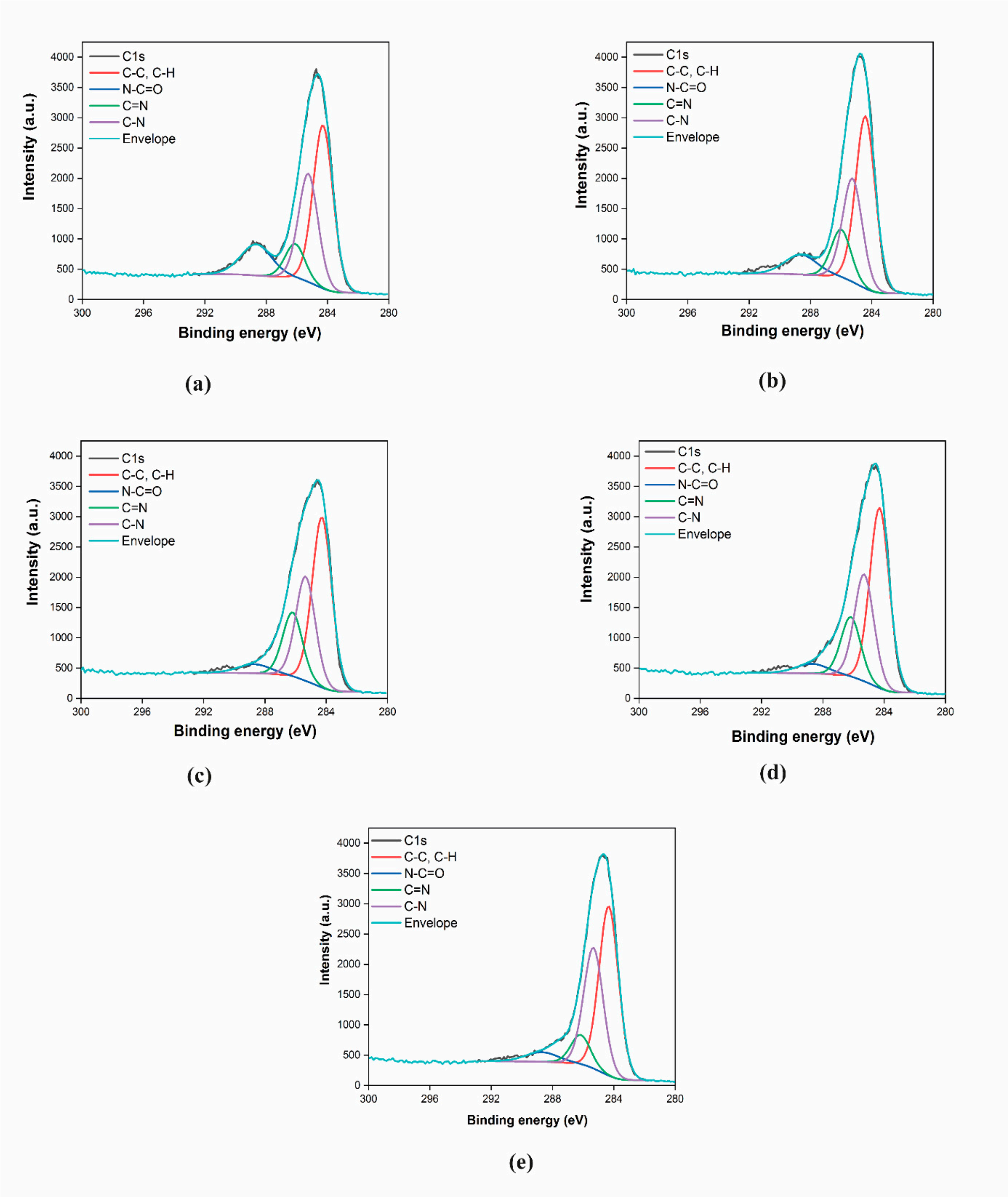

Figure S4. High resolution XPS spectra of C1s for U-PPD2 (**a**), U-PPD4 (**b**), U-PPD6 (**c**), U-PPD8 (**d**) and U-PPD16 (**e**).

Table S2. Elemental composition of U-PPD2, U-PPD4, U-PPD6, U-PPD8 and U-PPD16 by XPS.

Sample Name	C%	O%	N%
U-PPD2	69.11	7.64	23.24
U-PPD4	69.86	7.7	22.44
U-PPD6	71.54	11.76	16.7
U-PPD8	71.45	10.65	17.9
U-PPD16	71.76	10.52	17.72

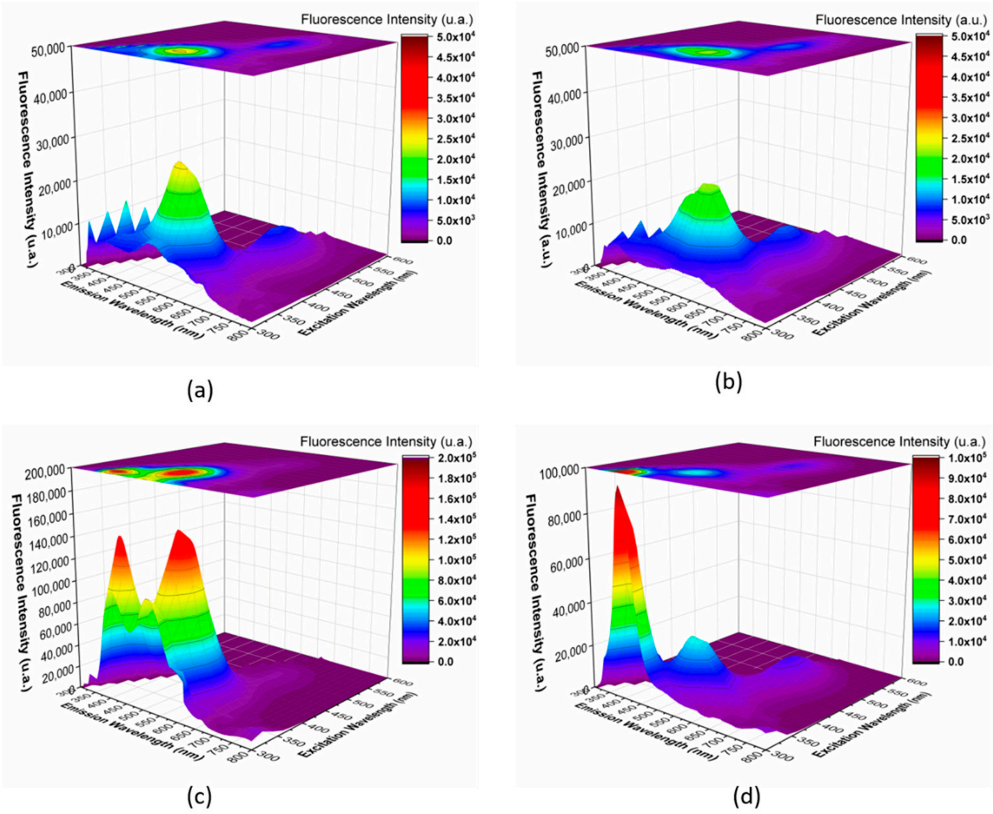

Figure S5. 2D fluorescence color map of D-PPD16 (**a**), PPD16 (**b**), U-PPD16 (**c**) and D-U-PPD16 (**d**).

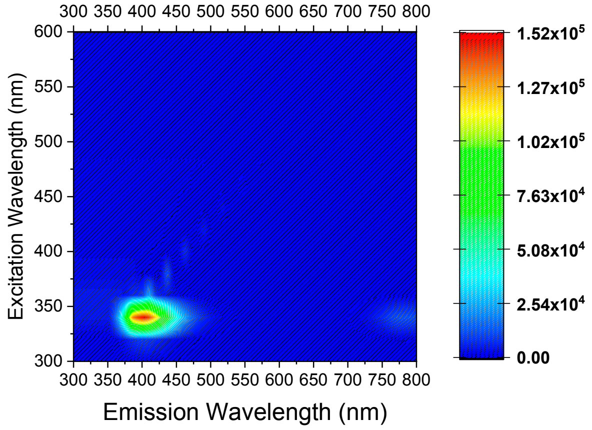

Figure S6. 2D fluorescence contour plot of p-phenylenediamine.

Table S3. Lifetime measurements of U-PPD2 (**a**) and U-PPD16 (**b**).

	**Lifetime (ns)**	**Amplitude**
$\mathbf{T_1}$	0.43	70.28 %
$\mathbf{T_2}$	3.27	29.72 %
**Average**	0.585	

(**a**)

	**Lifetime (ns)**	**Amplitude**
$\mathbf{T_1}$	0.55	6.66 %
$\mathbf{T_2}$	3.21	41.37 %

**T_3**	6.15	51.97 %
**Average**	3.01	

(**b**)

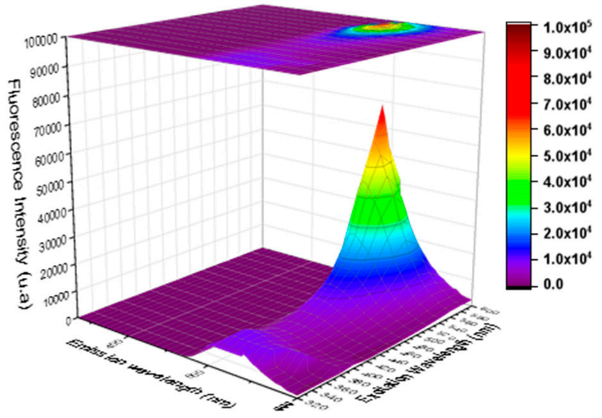

Figure S7. 2D fluorescence color map of Bandrowski's Base synthesized according to a previous literature method.

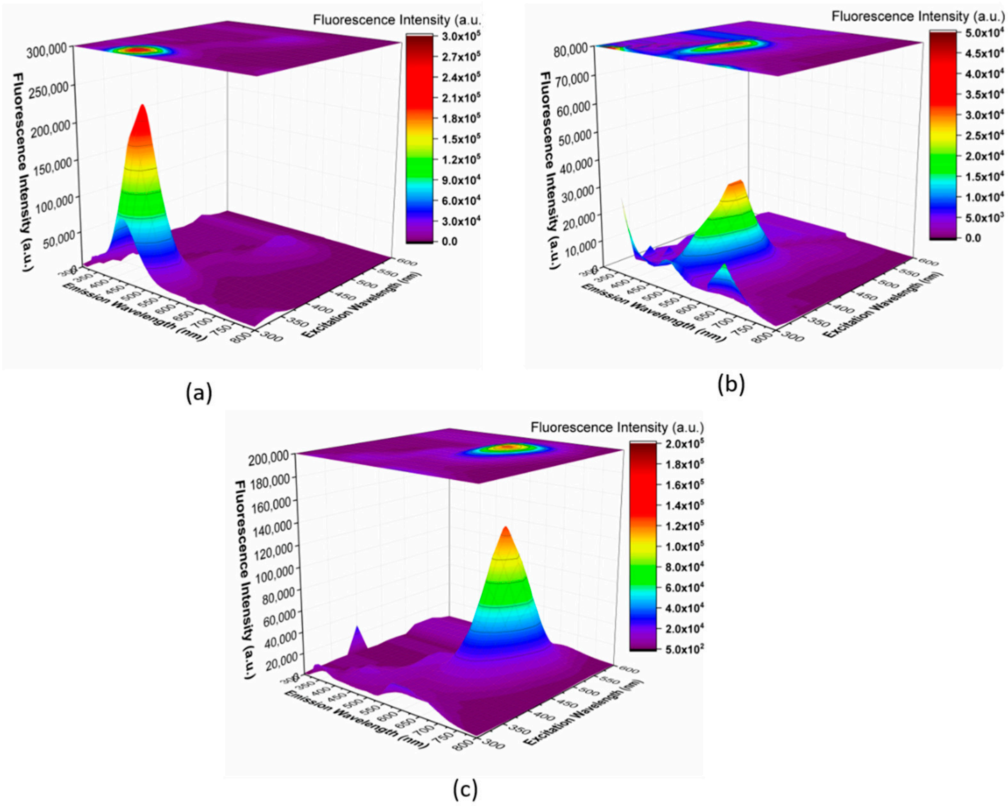

Figure S8. 2D fluorescence color map of isolated compounds with emission centers located at λ_{exc}= 340 nm, λ_{em} = 402 nm (**a**), λ_{exc} = 420 nm, λ_{em} = 511 nm (**b**) and λ_{exc} = 520 nm, λ_{em} = 606 nm (**c**).

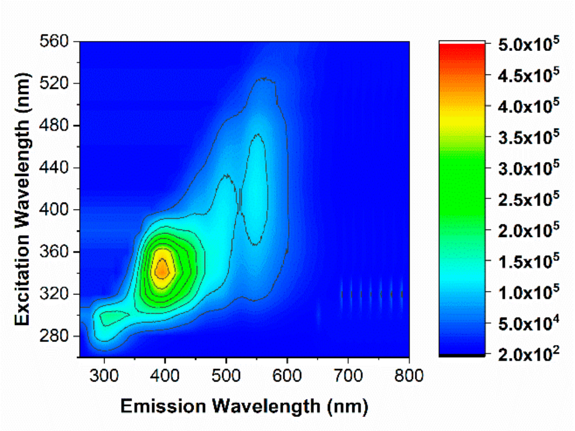

Figure S9. 2D fluorescence contour plot of U-PPD16 embedded into Mowiol 40-88 (PVA).

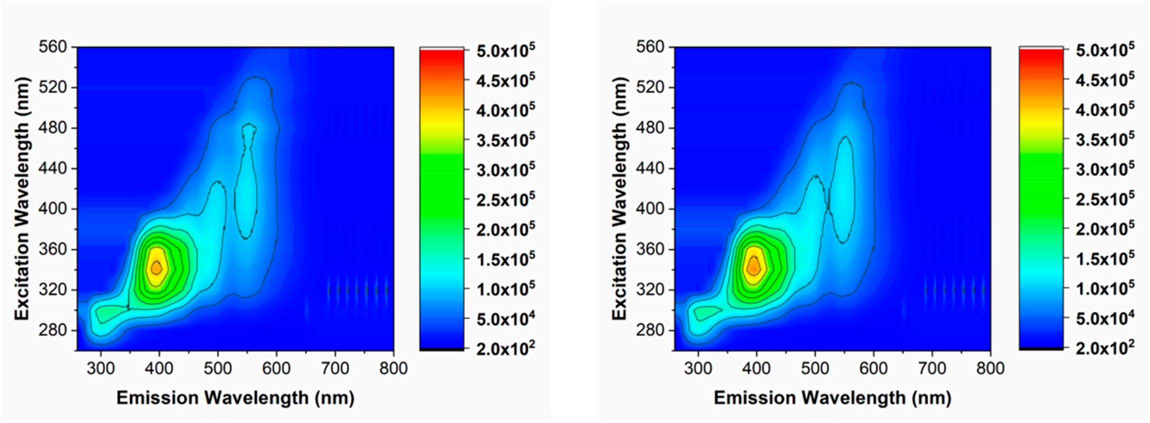

(a) (b)

Figure S10. 2D flourescence contour plot of U-PPD16 embedded into Mowiol 40-88 under ambient condition (**a**) and 21 kPa O_2 (**b**).

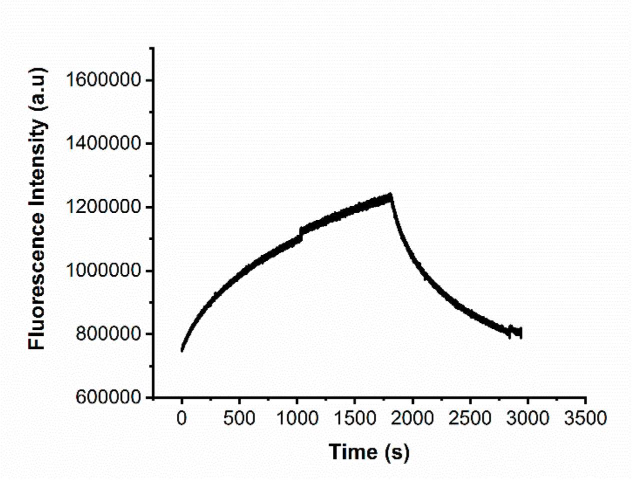

Figure S11. Response time of the third emission center of U-PPD16 embedded into Mowiol 40-88.
